# Possible Involvement of Tissue Plasminogen Activator/Brain-Derived Neurotrophic Factor Pathway in Anti-Depressant Effects of Electroacupuncture in Chronic Unpredictable Mild Stress-Induced Depression in Rats

**DOI:** 10.3389/fpsyt.2020.00063

**Published:** 2020-02-20

**Authors:** Tong Luo, Huiling Tian, Hongtao Song, Jun Zhao, Ai Liya, Yumin Fang, Junhui Mou, Zhigang Li, Saiyin Chaoketu

**Affiliations:** ^1^Department of Acupuncture, Moxibustion and Tuina, Beijing University of Chinese Medicine, Beijing, China; ^2^Department of Traditional Chinese Medicine, Inner Mongolia People’s Hospital, Hohhot, China; ^3^Graduate School, Inner Mongolia Medical University, Hohhot, China; ^4^Department of Wu-Liao and Rehabilitation, Inner Mongolia International Mongolian Hospital, Hohhot, China

**Keywords:** electroacupuncture, hippocampus, raphe nuclei, tPA, BDNF, depression

## Abstract

**Objective:**

Using a rat model of chronic unpredictable mild stress (CUMS), to investigate the effects of electroacupuncture (EA) on the tissue plasminogen activator (tPA)/brain-derived neurotrophic factor (BDNF) pathway.

**Methods:**

Sixty male Sprague–Dawley rats were randomly divided into four groups: normal, model, fluoxetine (fluox), or EA. Experimental groups were subjected to 28 d of CUMS modeling. One hour after CUMS, the fluox and EA groups were treated with ﬂuox and a 20 min EA intervention, respectively. Depressive-like behaviors were assessed by open field and sucrose preference tests. After the rats were sacrificed, brains were dissected and processed using hematoxylin and eosin (HE) staining to observe changes in the morphology and quantity of neurons in the hippocampal *cornu ammonis* 3 area. Western blot and real-time polymerase chain reaction (PCR) demonstrated the effects of EA on the tPA/BDNF pathway-related molecules in the hippocampi and raphe nuclei.

**Results:**

Compared to the model group, the number of horizontal and vertical movements and the percentage of sucrose consumption in the EA groups were significantly increased (P < 0.01). Compared to the model group, HE staining showed that the hippocampal neurons in the EA and fluox groups were arranged neatly, with rich layers and complete cell structures. The Western blot and real-time PCR showed that the levels of tPA, BDNF, tropomyosin receptor kinase B, and BDNF micro RNA (mRNA) in the hippocampi of the EA group were higher than in the model group (P < 0.01, P < 0.01, P < 0.05, P < 0.01, respectively). The content of p75NTR, proBDNF, and tPA mRNA in the hippocampi of the EA group displayed no significant differences compared to the model group. The tPA mRNA content in the raphe nuclei of the EA group was higher than in the model group (P < 0.01), and the BDNF content in the raphe nuclei was lower than in the model group (P < 0.05). There were no significant differences in tPA and BDNF mRNA between the EA and model groups.

**Conclusion:**

EA may reverse depressive-like behaviors in CUMS, which may be related to the tPA/BDNF pathway in the hippocampus.

## Introduction

Depression is a common mental illness that is primarily associated with persistent mood dysfunction and is one of the leading causes of disability worldwide (1). As an important public-health problem, its high burden and disability have a large impact on individuals, families, and society (2).

First-line antidepressants, such as selective serotonin reuptake inhibitors, have notable shortcomings including limited therapeutic efficacy, significant time lag for treatment response, and various side effects (3, 4). Acupuncture has been considered effective in the treatment of depression as it alleviates depressive symptoms in rats who have experienced maternal separation. Moreover, electroacupuncture (EA) has immediate and short-term effects in alleviating chronic pain, autonomic dysfunction, and mood disorder symptoms by modulating a distributed network of brain areas (5, 6).

The hippocampus is an important part of the limbic system as it participates in cognitive function and is critical in emotion regulation (7). Additionally, previous studies have found that impairments to the raphe nuclei have been found in patients with varying severities of depression using transcranial sonography and the structure’s echogenicity in patients with unipolar depression is typically lower than patients with Parkinson’s disease or healthy controls (8, 9). While the underlying etiology and pathophysiology of depression remains unclear, these studies suggest that changes to the neurons in the hippocampus and raphe nuclei are important in the development and treatment of depression.

Studies have found that the pathogenesis of depression and the mechanisms underlying antidepressant therapeutic action are related to the tissue plasminogen activator (tPA)/brain-derived neurotrophic factor (BDNF) lysis pathway (10). The combination of tPA, BDNF, tropomyosin receptor kinase B (TrkB), proBDNF, and p75 neurotrophin receptor (p75NTR) have been suggested as a diagnostic biomarker panel to diagnose depression (10). BDNF is initially synthesized intracellularly in the form of the glycosylation precursor protein proBDNF, which is encoded by the *BDNF* gene on human chromosome 11, band p13 (11). During the *in vitro* culture, proBDNF is converted to mature BDNF in the *trans*-Golgi network and/or immature secretory vesicles (12). Several studies have found that extracellularly, proBDNF can be cleaved into mature BDNF by the extracellular protease tPA/plasmin cascade (13, 14). These studies indicate that there are two cleavage pathways for proBDNF.

In addition to the biological activity of mature BDNF, proBDNF can also lead to depression by inducing peripheral neuronal apoptosis, while both pro- and mature BDNF exert opposite biological effects *in vivo* (15). Both proBDNF and BDNF have their own preferred cognate receptors; proBDNF causes neuronal apoptosis by binding to the p75NTR receptor and BDNF maintains neuronal survival by preferentially activating the TrkB receptor (16).

tPA is important in dividing proBDNF into mature BDNF (17). When tPA is knocked out in the hippocampus, it can lead to depression and anxiety-like behaviors in adult mice, while the injection of tPA over-expressing vectors in the hippocampus can reverse these effects (18).

Since EA has been found to be effective in the treatment of depression, there is a large amount of research on its mechanism of action in depression; however, specific mechanisms have not been fully elucidated. In the present study, we investigated the effects of EA on tPA/BDNF pathway-related molecules in the hippocampus and raphe nuclei to better understand its mechanisms in the treatment of depression, thereby helping provide a theoretical basis for the clinical application of EA.

## Materials and Methods

### Experimental Animals

Sixty male Sprague–Dawley specific-pathogen-free rats (200–220 g; 2–3 months old) were provided by Beijing Weitong Lihua Experimental Animal Technology Co. Ltd. (license no.: SCXK 2016-0006; Beijing, China) and permitted to acclimatize for 1 week before experimental procedures began. All experimental procedures were approved by the ethics committee of the Beijing University of Chinese Medicine in accordance with the Guiding Opinions on Treating Experimental Animals issued by the Ministry of Science and Technology of the People’s Republic of China (protocol no.: BUCM-4-2018062201-2073). Rats were randomly assigned to four groups (n = 15 per group): normal, model, fluoxetine (fluox), and EA.

### Main Experimental Reagents and Instruments

Anti-tPA (Novus Biologicals, Centennial, CO, USA), anti-BDNF (Novus Biologicals), anti-TrkB (Abcam, Cambridge, MA, USA), anti-ProBDNF (Novus Biologicals), anti-p75NGF receptor (Abcam), anti-GAPDH (Abcam), fluoxetine hydrochloride capsule (French Patheon, Bourgoin, France), TRIzol kit (Invitrogen, Carlsbad, CA, USA), polymerase chain reaction (PCR) primer (Bio-Bioengineering Shanghai Co. Ltd., Shanghai Shi, China), M-MLV reverse transcription kit (Promega, Madison, WI, USA), real-time PCR amplification kit (Beijing Zhongyuan Leading Technology Co. Ltd., Beijing, China), stabilized flow electrophoresis system (Bio-Rad Laboratories, Hercules, CA, USA), semi-dry transfer instrument (Bio-Rad Laboratories), real-Time PCR instrument (ABI 7500; Thermo Fisher Scientific, Waltham, MA, USA), electronic balance (BS224S; Satorius, Göttingen, Germany), nucleic acid UV spectrometry photometer (Eppendorf, Hamburg, Germany), and an open field test box (self-made; 60 cm × 60 cm × 40 cm).

### Establishment of Chronic Unpredictable Mild Stress Model

The CUMS rat model for depression is considered to be ideal for elucidating stress-induced depression. In our study, CUMS procedure was performed as described previously (19, 20) with minor modifications. Other than those in the normal group, rats were all orphaned and subjected to chronic unpredictable mild stress (CUMS) for 28 days. A total of seven different stressors were used: 4°C cold water swimming (5 min), day and night reversal (24 h), food deprivation (24 h), water deprivation (24 h), tail pinching (3 min), damp sawdust (24 h), and restraint (3 h). The stimulation was alternated every other day and each stimulus appeared no less than four times.

### Electroacupuncture and Fluoxetine Administration

**Figure 1:**
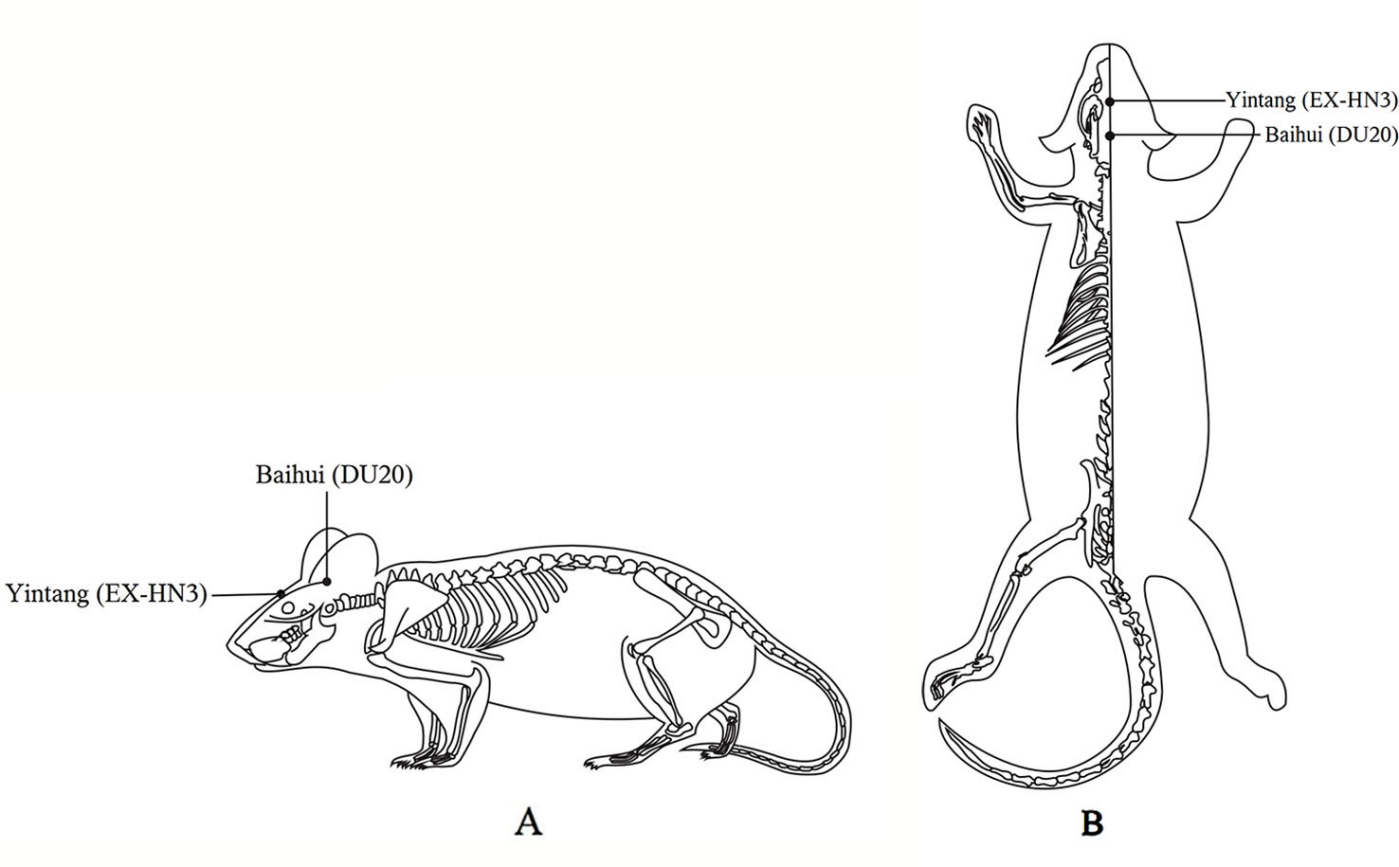
Locations on the rat body where electroacupuncture was applied. **(A)** The lateral view. **(B)** The coronal view.

Body weight and behavioral performance were measured before and after CUMS. The EA group received 20 min of EA (2 Hz, 2 mA) daily at the Baihui (DU20) and Yintang (EX-HN3) acupoints 1 h after CUMS. The acupoints were determined by the “Atlas of Acupuncture Point for Experimental Animals” formulated by the Experimental Acupuncture Research Association of the National Acupuncture Society in China (**Figure 1**). The needle tip placed at “Baihui” was towards the hind head and the needle tip placed at “Yintang” pointed to the nasal tip. The two needle handles were separated by a cotton ball to prevent short circuiting. Finally, the fluox group was intragastrically administered ﬂuoxetine (2 mg/kg; diluted with saline to 1 ml; the 2 mg dose of fluoxetine refers to fluoxetine hydrochloride alone) 1 h after modeling daily (21).

### Behavioral Tests, Histology, and Identification of Biomarkers

#### Sucrose Preference Test

Depressive-like phenotype was assessed using the sucrose preference test (SPT) as described previously (22, 23). In the preliminary stages of the experiment, the animals were trained to adjust to the sugary drinking water in a quiet environment. Two identical water bottles were placed in each cage. In the first 24 h, both bottles were equal weights consisting of 1% sucrose water; in the second 24 h, one bottle contained 1% sucrose water and the other bottle was the same weight with only pure water. Following this, rats were water deprived for 24 h. In the test phase, each rat was given a bottle of 1% sucrose water (250 g bottle weight) and a bottle of purified water (250 g bottle weight). After 24 h, the consumption of sucrose water and purified water was measured to calculate sucrose preference percentage [SPP = sucrose solution consumption/(sucrose solution consumption + water consumption) × 100%].

#### Open Field Test

The open field test (OFT) was conducted on the day before and after CUMS to assess the exploratory activity level (24). Experimental animals were placed in an open box (60 cm × 60 cm × 40 cm), in which the floor was divided into 25 equal squares with the walls painted black. The number of times the rats passed through the bottom surface (three claws were all entered into the square) was counted as the number of horizontal activity and the number of times the rats were erect (two front paws vacating or climbing the walls of box) was counted as vertical activity. The number of horizontal movements and the number of vertical movements were measured once per rat for 3 min. The whole process of the test was recorded with a video recorder and the test environment was kept quiet during the recording. After each rat completed the test, the box was sterilized with 75% alcohol and the rat feces in the box were cleaned to avoid affecting the behavior of the next rat.

#### Hematoxylin and Eosin (HE) Staining

After the rats were anesthetized with 10% chloral hydrate, the chest and right atrial appendage were cut open and the left ventricle was flushed with normal saline and perfused with 4% paraformaldehyde. After perfusion, rats were decapitated and the brain was dissected, placed in 4% paraformaldehyde, and then fixed for an additional 24 h. Next, the specimens were removed and embedded in paraffin. Sections were stained in HE solutions and then mounted with coverslips. Last, the morphology and quantity of nerve cells in the hippocampal CA3 area were observed under a 20**×** microscope.

#### Western Blot

Hippocampus and raphe nuclei samples (50 mg) were lysed, homogenized, and centrifuged at 10,000*g* for 10 min at 4°C. Bicinchoninic acid assay was used to determine the concentration of proteins in the supernatant, which were subjected to electrophoresis and then transferred to polyvinylidene fluoride membranes. The membranes were blocked with 5% TBST skimmed milk and shook at room temperature for 60 min. After the block, antibodies (BDNF 1:1,000; tPA 1:1,000; proBDNF 1:500; p75NTR 1:1,000; TrkB 1:1,000; GAPDH 1:5,000) were added and diluted with the appropriate rinse. The membranes were incubated in horseradish peroxidase conjugated secondary antibody (BDNF 1:1,000; tPA 1:2,000; proBDNF 1:1,000; p75NTR 1:1,000; TrkB 1:2,000; GAPDH 1:10,000) for 1 h at room temperature. Using the Integrated Performance Primitives software (Intel, Santa Clara, CA, USA) gray-scale analysis was performed on the target strip of the scanned images.

#### Real-Time Polymerase Chain Reaction

Total micro RNA (mRNA) was extracted from the hippocampus and raphe nuclei using TRIzol reagent according to the kit instructions. Real-time PCR program settings were performed according to the manufacturer’s instructions. See **Table 1** for primer sequence references.

**Table 1:** Primer sequence references (forward and reverse) for *BDNF*, *tPA*, and *GAPDH*.

Gene	Forward primer	Reverse primer
*BDNF*	5'-TGCTGGATGAGGACCAGAAG-3'	5'-TTCCTCCAGCAGAAAGAGCA-3'
*tPA*	5'-AGAGTGTGAGCTTTCTGGCT-3'	5'-TGGACGGATACAGTCTGACG-3'
*GAPDH*	5'-CAACTCCCTCAAGATTGTCAGCAA-3'	5'-GGCATGGACTGTGGTCATGA-3'

Relative gene expression levels were analyzed by relative quantitative 2^−ΔΔCT^ method.

#### Statistical Analysis

Statistical data were analyzed using SPSS statistical software (version 22.0; IBM Corporation, Armonk, NY, USA). Data results are expressed as mean ± standard deviation (x ± s). Multiple comparisons were analyzed by one-way analysis of variance (ANOVA). When the variance was equal, the least significant difference method was used; otherwise, Dunnett’s T3 was used. P < 0.05 was considered statistically significant.

## Results

### Body Weight and Behavioral Results

**Figure 2:**
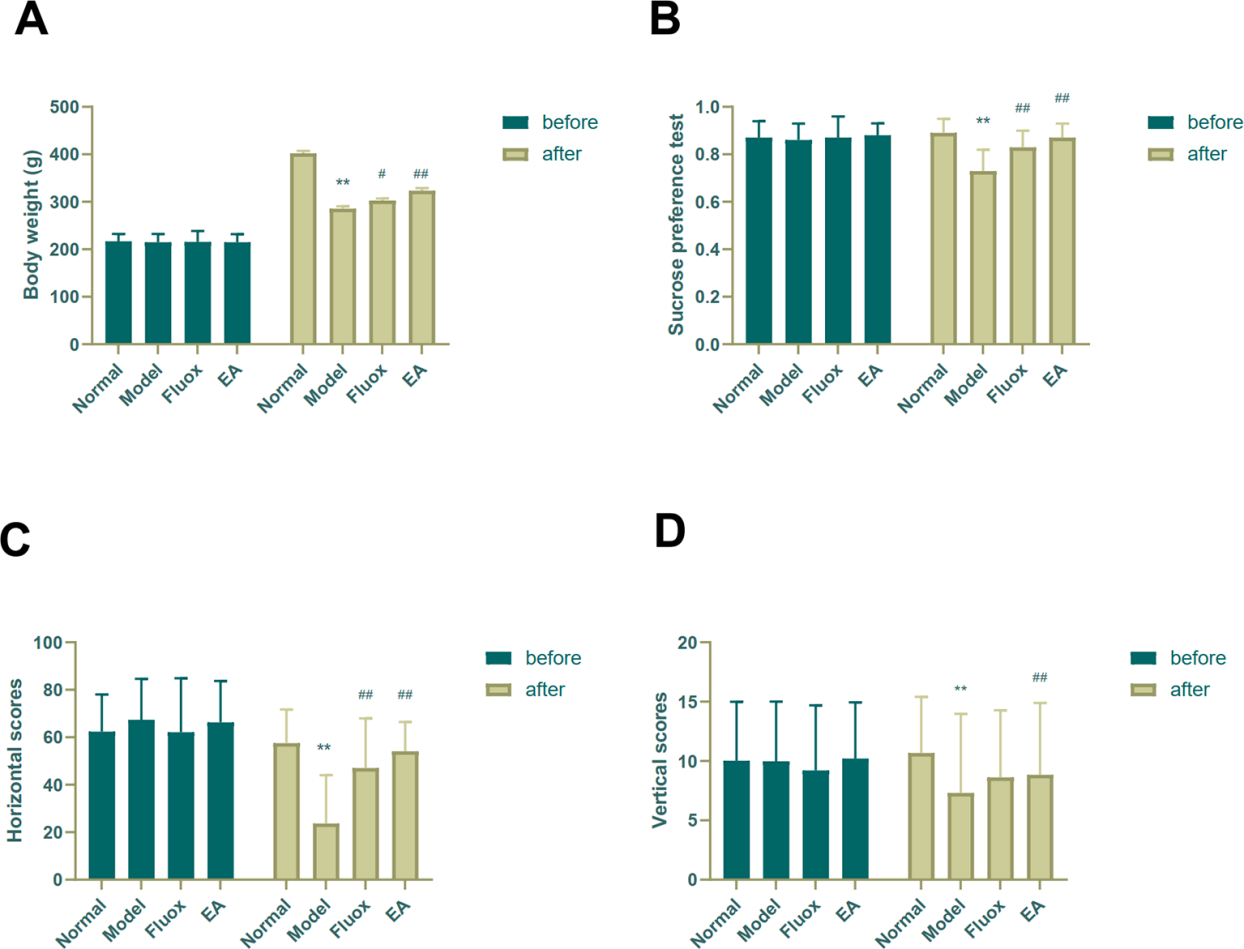
Effects of electroacupuncture on body weight and behavioral performance before and after chronic unpredictable mild stress in rats. The results of body weight **(A)**, sucrose preference percentage **(B)**, horizontal scores **(C)** of open field test, and vertical scores **(D)** of open field test were measured and calculated (data are presented as mean ± standard error of measurement, n = 15 per group. **P < 0.01 versus normal; ^#^P < 0.05, ^##^P < 0.01 versus model). Fluox, fluoxetine group; EA, electroacupuncture groups.

Depressive-like symptoms were evaluated using body weight, SPT, and OFT. One-way ANOVAs showed significant differences in body weight (n=15; F = 53.825, P < 0.001; **Figure 2A**), sucrose preference (F = 14.469, P < 0.001; **Figure 2B**), and horizontal (F = 11.551, P < 0.001; **Figure 2C**) and vertical (F = 9.357, P < 0.001; **Figure 2D**) scores of the OFT in the four groups after CUMS. Four weeks of CUMS procedures caused a significant decrease in weight gain (P < 0.01; **Figure 2A**), but EA (P < 0.01; **Figure 2A**) or fluox (P < 0.01; **Figure 2A**) could reverse this change. The CUMS rats developed lower sucrose preference percentage in the SPT (P < 0.01; **Figure 2B**), but EA (P < 0.01; **Figure 2B**) or fluox (P < 0.01; **Figure 2B**) could reverse this. In the OFT, horizontal and vertical scores were significantly different between the EA and model groups. CUMS rats demonstrated less horizontal (P < 0.01; **Figure 2C**) and vertical (P < 0.01; **Figure 2D**) activity, whereas the EA group demonstrated more horizontal (P < 0.01; **Figure 2C**) and vertical (P < 0.01; **Figure 2D**) movements. Additionally, the fluox group demonstrated increased horizontal (P < 0.01; **Figure 2C**) movements, but not vertical (P >0.05; **Figure 2D**).

### HE Staining Results of Hippocampal CA3 Area

**Figure 3:**
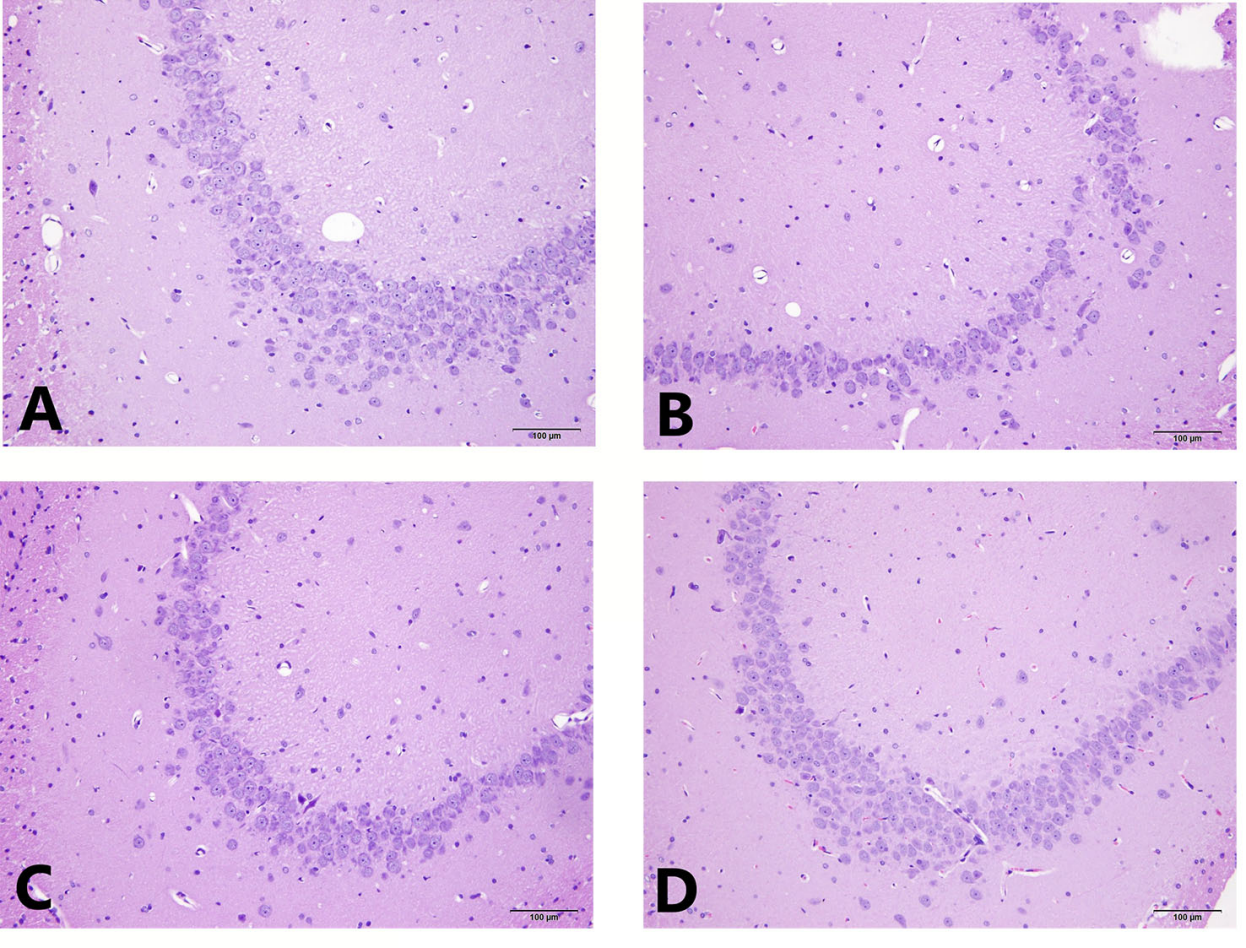
HE staining of hippocampal CA3 area. **(A)** is the normal group; **(B)** is the model group; **(C)** is the fluoxetine group; and **(D)** is the electroacupuncture group.

The results of the light microscopic observation of the hippocampal CA3 showed that these neurons in the normal group were arranged regularly, the cell layer was rich, the cell membrane and cytoplasmic morphology were intact, and the nucleus was round and large. In the model group, the number of hippocampal neurons was significantly reduced, the structure was disordered, the neuron cells were atrophied, the cell shape was irregular, the cell gap became larger, the nucleolus was not obvious, and some neurons were vacuolated. Compared with the model group, the hippocampal cells in the EA and the fluox groups were more regular, the cells were arranged neatly, and the nucleolus was clearer. See **Figure 3** for HE staining of the hippocampal CA3 area.

### Protein Levels of BDNF, TrkB, tPA, proBDNF, and p75NTR in the Hippocampus

**Figure 4:**
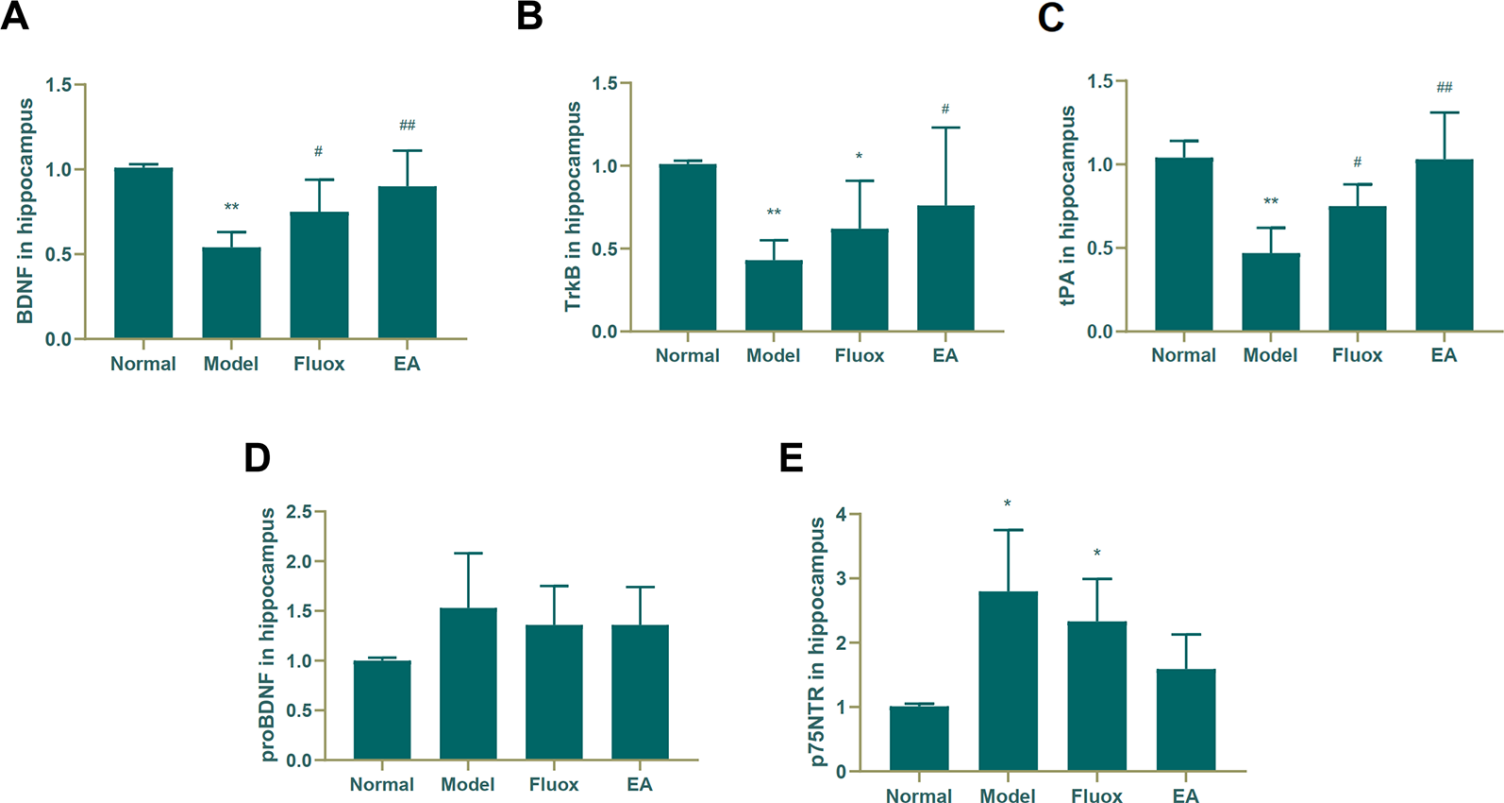
Effects of electroacupuncture on the production of brain-derived neurotrophic factor (BDNF) **(A)**; tropomyosin receptor B (TrkB) **(B)**; tissue plasminogen activator (tPA) **(C)**; proBDNF (**D**), and p75NTR **(E)** in the hippocampus. Their contents were measured and calculated (data are presented as mean ± standard error of the mean, n = 6. *P < 0.05, **P < 0.01 versus normal; ^#^P <0.05, ^##^P <0.01 versus model).

To determine if EA had an effect on tPA/BDNF pathway-related proteins, BDNF, TrkB, tPA, proBDNF, and p75NTR were measured. The concentrations of BDNF (F = 11.171, P < 0.01; **Figure 4A**), TrkB (F = 4.631, P = 0.013; **Figure 4B**), and tPA (F = 14.147, P < 0.01; **Figure 4C**) in the hippocampus significantly differed between the four groups. In the model group, CUMS significantly decreased BDNF (P < 0.01; **Figure 4A**), TrkB (P < 0.01; **Figure 4B**), and tPA (P < 0.01; **Figure 4C**) protein levels in the hippocampus, but EA and fluox could reverse these changes (P < 0.01 and P < 0.05, respectively); however, the concentration of proBDNF (F = 1.933, P = 0.157) in the hippocampus did not significantly differ between the four groups (see **Figure 4D**). **Figure 4E** demonstrates that the concentration of P75NTR (F = 9.259, P < 0.01) in the hippocampus were statistically significantly different between the groups. Both CUMS and fluox markedly elevated the expression of P75NTR in the hippocampus of CUMS rats (P < 0.05; **Figure 4E**).

### Protein Levels of BDNF and tPA in the Raphe Nuclei

**Figure 5:**
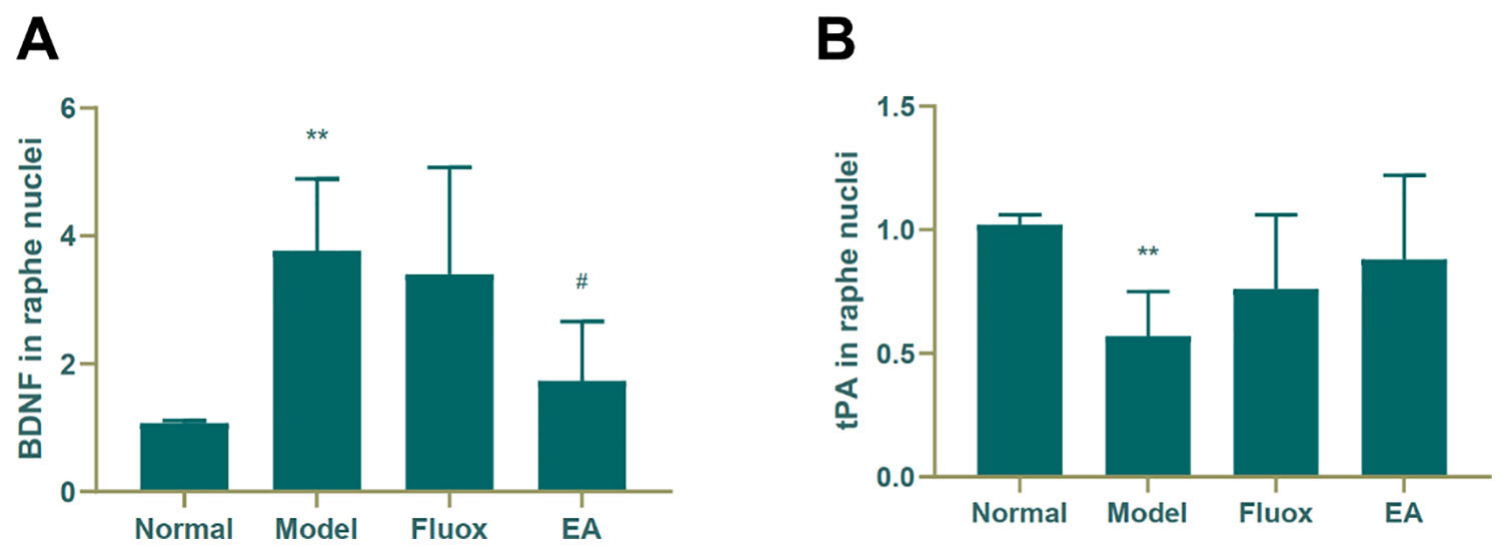
Effects of electroacupuncture on the production of brain-derived neurotrophic factor (BDNF) **(A)**; and tissue plasminogen activator (tPA) **(B)**; in the raphe nuclei (data are presented as mean ± standard error of mean, n = 6. **P < 0.01 versus normal; ^#^P < 0.05 versus model).

**Figure 5A** shows that the concentration of BDNF (F = 8.221, P < 0.01) in the raphe nuclei significantly differed between the four groups. CUMS elevated BDNF protein levels in the model group (P < 0.01; **Figure 5A**), while a decrease was seen in the BDNF protein level in the raphe nuclei of CUMS rats (P < 0.05; **Figure 5A**). In contrast, CUMS decreased the expression of tPA (P < 0.01; **Figure 5B**) compared to the normal group, but neither EA nor fluox restored its expression.

### Concentration of BDNF and tPA mRNA in the Hippocampus and Raphe Nuclei

**Figure 6:**
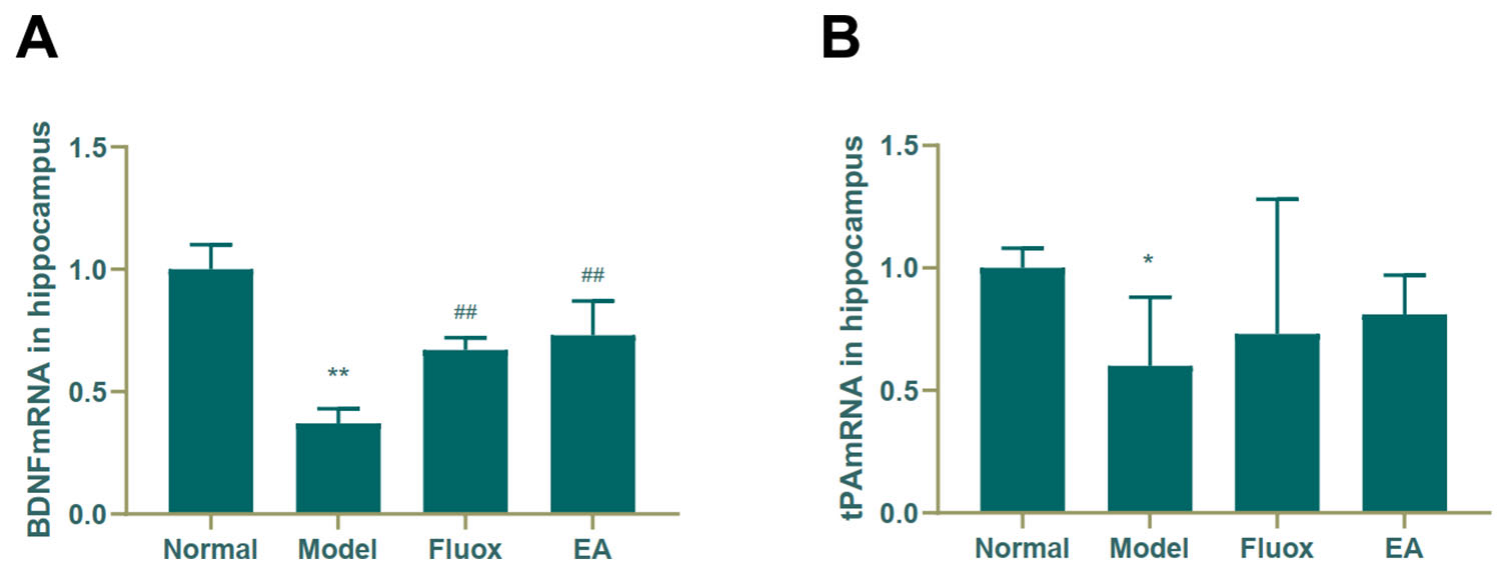
Effects of electroacupuncture on the level of brain-derived neurotrophic factor (BDNF) micro RNA (mRNA) **(A)**; and tissue plasminogen activator (tPA) mRNA **(B)** in the hippocampus (data are presented as mean ± standard error of mean, n = 6. *P < 0.05, **P < 0.01 versus normal; ^##^P < 0.01 versus model).

**Figure 7:**
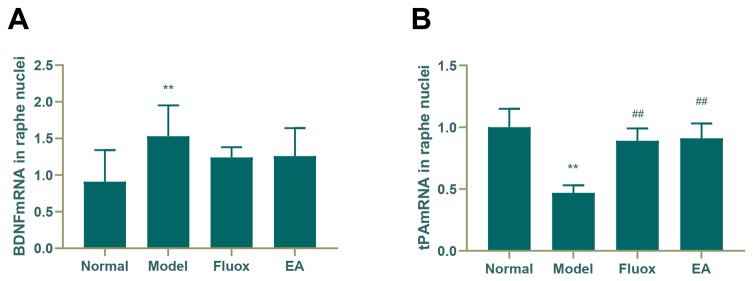
Effects of electroacupuncture on the level of brain-derived neurotrophic factor (BDNF) micro RNA (mRNA) **(A)**; and tissue plasminogen activator (tPA) mRNA **(B)** in the raphe nuclei (data are presented as mean ± standard error of mean, n = 6. **P < 0.01 versus normal; ^##^P < 0.01 versus model).

A one-way ANOVA showed that expression of BDNF mRNA (F = 42.488, P < 0.01; **Figure 6A**) in the hippocampus and tPA mRNA (F = 25.929, P < 0.01; **Figure 7B**) in the raphe nuclei were statistically significantly different between the four groups. In the model group, CUMS significantly decreased BDNF mRNA (P < 0.01; **Figure 6A**) and tPA mRNA (P < 0.05; **Figure 6B**) levels in the hippocampus. While in the hippocampus, EA and fluox increased the level of BDNF mRNA (P < 0.01), neither EA nor fluox restored tPA mRNA (P >0.05; **Figure 6B**). Unexpectedly, CUMS elevated BDNF mRNA levels in the model group (P < 0.01; **Figure 7A**).

## Discussion

CUMS model can induce the core symptoms of depression in rats by simulating unpredictable life stress and has been widely used in the induction of depressive-like behaviors in rodents (25, 26). The OFT evaluated changes in autonomy and curiosity behaviors in rats, while the SPT evaluated the degree of anhedonia.

Behavioral test results showed that the horizontal and vertical scores on the OFT and SPT in the EA and fluox groups were significantly improved. These results indicate that EA and fluox could significantly mitigate depressive symptoms in rats and significantly improve their autonomy and spatial exploration. Both EA and fluox adequately reduced the level of anhedonia in the depressed rats; however, the EA group demonstrated better improvements than the fluox group.

The Western blot and real-time PCR showed that EA could significantly increase the concentrations of tPA, BDNF, TrkB, and BDNF mRNA in the hippocampus. There was no statistical difference in the concentration of proBDNF and p75NTR in the hippocampus of the EA group, but there was a notable downward trend. In the raphe nuclei of the EA group, the *tPA* mRNA concentration was increased, while the *BDNF* was decreased.

As earlier described, BDNF is initially synthesized intracellularly in the form of proBDNF, which is encoded by the *BDNF* gene on human chromosome 11 (11). The content of BDNF, BDNF mRNA, and tPA in the hippocampi of rats in the EA group was higher than that in model group; however, there was no significant difference in hippocampal proBDNF concentration compared to model group. Therefore, we believe that EA promotes the conversion of proBDNF to BDNF by increasing the content of tPA in hippocampus, which elicits antidepressant effects.

In the EA group, the protein concentrations of BDNF and TrkB in the hippocampus were increased compared to the model group, while the concentrations of proBDNF and p75NTR protein had no statistically significant difference. It is known that BDNF and proBDNF act by binding different high affinity receptors in organisms (27, 28). Based on these results, we speculate that EA elicits antidepressant effects by increasing the concentrations of BDNF and TrkB to maintain neuronal survival.

The content of BDNF in the raphe nuclei was decreased in the EA group compared to the model group. Moreover, BDNF mRNA in the raphe nuclei was not significantly different compared to the model group, but there was a decreasing trend. These suggest that tPA levels may be decreasing; however, these increased tPA mRNA levels were inconsistent with our hypotheses. This may be related to the distribution of different neuronal nuclei in the raphe nuclei. The raphe nuclei are distributed along the midline of the brainstem, can be divided into several neuronal nuclei based on the distribution of cellular structures (29, 30). Studies have found that the raphe magnus nucleus and the raphe pallidus nucleus in the raphe nuclei have a dense reciprocal connection with BDNF neurons (31). There is also literature that has demonstrated that the raphe nuclei, especially the dorsal raphe nucleus (DRN), is the main distribution site of serotonin neurons in the brain (32, 33). The DRN is an origin of serotonergic projections to the forebrain and is considered to be an important component of the brain circuit that regulates anxiety and depression-related behaviors (33). Therefore, we suspect that the mechanisms underlying the treatment of depression using EA are complex, and that the targets or pathways of the varying brain regions or neurons differ. In the present study, we found that EA had little effect on the tPA/BDNF pathway in the raphe nuclei. The relationship between the tPA/BDNF pathway in the raphe nuclei and other pathways, as well as the relationships of the competition and inhibition between the tPA/BDNF pathway and other pathways need to be studied further.

In summary, EA improved depressive-like symptoms in CUMS model rats. We believe that this mechanism may be related to the tPA/BDNF pathway in the hippocampus.

## Data Availability Statement

The raw data supporting the conclusions of this article will be made available by the authors, without undue reservation, to any qualified researcher.

## Ethics Statement

The animal study was reviewed and approved by the ethics committee of the Beijing University of Chinese Medicine in accordance with the Guiding Opinions on Treating Experimental Animals issued by the Ministry of Science and Technology of the People’s Republic of China.

## Author Contributions

All authors were involved in designing the study and assisted in data collection. TL and HT contributed to the interpretation of results and writing of manuscript. HS, JZ, AL, YF, and JM performed the data analyses. ZL and SC made critical revisions and approved the final manuscript.

## Funding

This research was supported by grants from the National Natural Science Foundation of China (No. 81760909, XL) and Natural Science Foundation of Inner Mongolia Autonomous Region (No. 2017BS0815, XL).

## Conflict of Interest

The authors declare that the research was conducted in the absence of any commercial or financial relationships that could be construed as a potential conflict of interest.
